# Comparison of fast Fourier transform infrared spectroscopy biotyping with whole genome sequencing-based genotyping in common nosocomial pathogens

**DOI:** 10.1007/s00216-022-04270-6

**Published:** 2022-08-13

**Authors:** Ayza S. J. Teng, Patrick E. Habermehl, Robin van Houdt, Menno D. de Jong, Rosa van Mansfeld, Sébastien P. F. Matamoros, Ingrid J. B. Spijkerman, Maurits P. A. van Meer, Caroline E. Visser

**Affiliations:** 1 Department of Medical Microbiology, Amsterdam UMC Location University of Amsterdam, Meibergdreef 9, Amsterdam, The Netherlands; 2 Department of Medical Microbiology, Amsterdam UMC Location Vrije Universiteit Amsterdam, Boelelaan 1117, Amsterdam, The Netherlands; 3 Department of Medical Microbiology, Rijnstate Hospital, Arnhem, The Netherlands

**Keywords:** Fourier transform infrared spectroscopy, Whole genome sequencing, Bacterial typing, Bacteria

## Abstract

Early detection of bacterial transmission and outbreaks in hospitals is important because nosocomial infections can result in health complications and longer hospitalization. Current practice to detect outbreaks uses genotyping methods amplified fragment length polymorphism (AFLP) and whole genome sequencing (WGS), which are not suitable methods for real-time transmission screening of both susceptible and resistant bacteria. The aim was to assess the typing technique Fourier transform infrared (FTIR) spectroscopy as real-time screening method to discriminate large amounts of susceptible and resistant bacteria at strain level when there is no evident outbreak in comparison with the WGS reference. Isolates of past hospital outbreak strains of *Acinetobacter baumannii/calcoaceticus complex* (*n* = 25), *Escherichia coli* (*n* = 31), *Enterococcus faecium* (*n* = 22), *Staphylococcus aureus* (*n* = 37) and *Pseudomonas aeruginosa* (*n* = 30) were used for validation of FTIR. Subsequently, *Enterococcus faecalis* (*n* = 106) and *Enterococcus faecium* (*n* = 104) isolates from weekly routine screening samples when no potential outbreak was present were analysed. FTIR showed reproducibility and congruence of cluster composition with WGS for *A. baumannii/calcoaceticus complex* and *E. faecium* outbreak isolates. The FTIR results of *E. faecalis* and *E. faecium* isolates from routine samples showed reproducibility, but the congruence of cluster composition with WGS was low. For *A. baumannii/calcoaceticus complex* and *E. faecium* outbreak isolates, FTIR appears to be a discriminatory typing tool. However, our study shows the discriminatory power is too low to screen real-time for transmission of *E. faecium* and *E. faecalis* at patient wards based on isolates acquired in routine surveillance cultures when there is no clear suspicion of an ongoing outbreak.

**Supplementary Information:**

The online version contains supplementary material available at 10.1007/s00216-022-04270-6.

## Introduction

Early detection of bacterial transmission and outbreaks in hospitals is important because bacterial nosocomial infections can result in serious health complications, longer hospitalization of patients and increased hospital costs [1–3]. Current practice is to respond to hospital outbreak signals such as increased number of observed infections by clinicians or cultures of multi-resistant bacteria using strain typing methods, like amplified fragment length polymorphism (AFLP) or the current gold-standard whole genome sequencing (WGS) to detect transmission routes in hospital. Genotyping methods like WGS and AFLP detect isolate variation in the composition, structure and sequence of genetic material, which makes them excellent for discriminating between closely related bacteria strains with high resolution [4, 5]. However, the current practice to respond to hospital outbreaks has two limitations which hampers the implementation of adequate infection prevention measures and can result in considerable health complications and financial burden. First, the focus of outbreak signals is mainly on multi-resistant bacteria, although nosocomial infections are also caused by susceptible variants [6], thus missing potential transmission routes and outbreaks in hospitals. Second, the current strain typing methods are not usable for quick real-time screening of transmission routes of both resistant and susceptible bacteria when no evident outbreaks are present; AFLP is not suitable to type large amount of strains, and WGS is too expensive and time-consuming.

Hence, there is an unmet need for rapid and inexpensive bacterial typing methods to screen both resistant and susceptible bacteria in real time. Detecting the presence of possible transmission before an outbreak can spread allows timely implementation of adequate infection prevention measures. A potential screening typing method is the Fourier transform infrared (FTIR) spectroscopy-based IR biotyper by Bruker Daltonics GmbH, Bremen, Germany, which was designed as an efficient and early warning system for transmission and outbreaks [7]. FTIR spectroscopy is a cheap and fast spectrum-based biotyping technique which generates an overall biochemical fingerprint of the bacterial composition [8–13]. Several studies used FTIR spectroscopy for rapid bacterial typing of isolates from food, environmental and clinical samples [2, 9–14]. As most past studies used collections of resistant bacteria, it is unknown if FTIR spectroscopy is capable of discriminating large amounts of resistant as well as large amounts of susceptible isolates at strain levels when there is no evident outbreak. To use FTIR spectroscopy as a real-time typing tool, it must be able to type resistant as well as sensitive strains.

If FTIR spectroscopy is a reliable method, we can combine FTIR spectroscopy as a real-time high-throughput screening method for routine samples with high-resolution confirmation of the gold-standard genotyping WGS to enable rapid, reliable and cost-effective detection of the spread of bacteria. This can reduce hospital infections, and major outbreaks can be prevented.

### Objectives and design

The performance of FTIR typing as real-time high-throughput screening method was assessed in three steps. Firstly, to assess the reproducibility of FTIR typing for intra-assay variability, a collection of well-characterized isolates from 5 different species recovered during past hospital outbreaks was analysed for clusters by FTIR spectroscopy and repeated by preparing the isolates three times. The repeated results were analysed for congruence of cluster composition. Secondly, for the species for which FTIR typing showed reproducibility, the results were analysed for congruence of cluster composition with WGS to measure the discriminatory power of FTIR spectroscopy. Finally, to validate FTIR spectroscopy as real-time screening method, intensive care patient routine rectal samples were collected when no evident hospital outbreak was present. For this step, the species *E. faecium* and *E. faecalis* were chosen, because they are frequently found in intensive care patient rectal samples. The FTIR spectroscopy results from these routine samples were analysed for congruence of cluster composition with WGS. This step was only conducted if in the first two steps FTIR spectroscopy had reproducible results and showed congruence with WGS for the outbreak strains of *E. faecium*.

## Materials and methods

### Isolates

This study used two different isolate collections to assess the performance of FTIR typing. The first collection contained multiple strains, well-characterized by AFLP (Table 1), from isolates of various past hospital outbreaks of the following five species: *Acinetobacter baumannii/calcoaceticus complex* (*n* = 25), *Escherichia coli* (*n* = 31), *Enterococcus faecium* (*n* = 22), *Staphylococcus aureus* (*n* = 37) and *Pseudomonas aeruginosa* (*n* = 30) (for further information, see supplement A). The bacterial isolates were stored in glycerol peptone at − 80 °C until used for FTIR- and WGS-typing analysis. Species determination of all thawed isolates was verified using MALDI-TOF (matrix-assisted laser desorption ionization-time of flight, Bruker Daltonics GmbH, Bremen, Germany).

**Table 1:** The AFLP cluster results of the multiple-strain past-outbreak collection of common nosocomial species

Species/cluster number	Cluster 1	Cluster 2	Cluster 3	Cluster 4	Cluster 5	Cluster 6	Cluster 7	Cluster 8	Non-clustering isolates
A. baumannii	16 isolates	4 isolates	2 isolates	2 isolates					1 isolate
E. coli	3 isolates	2 isolates	3 isolates	2 isolates	2 isolates	2 isolates	2 isolates	7 isolates	7 isolates
E. faecium	11 isolates	7 isolates	2 isolates						2 isolates
S. aureus	11 isolates	5 isolates	3 isolates	3 isolates					15 isolates
P. aeruginosa	9 isolates	4 isolates	2 isolates	2 isolates	2 isolates				10 isolates

For the second collection, isolates from weekly routine rectal screening samples were collected from intensive care units (ICU) patients when no potential outbreak was present during 2 consecutive weeks in October 2020. The routine samples were collected from patients who received selective decontamination of the digestive tract (SDD) at the Amsterdam University Medical Centers ICU. The species selected for this collection were *E. faecium* and *E. faecalis*, because they are frequently found in intensive care patient rectal samples. The *E. faecium* and *E. faecalis* isolates would only be collected if the FTIR spectroscopy results of the *E. faecium* outbreak isolates were reproducible and had congruence of cluster composition with WGS. If *E. faecium* and/or *E. faecalis* were present, three isolates per patient sample were collected. The species identification of the isolates was determined by MALDI-TOF mass spectrometry. The isolates were stored in Blood Bijoux tubes at room temperature before used for FTIR- and WGS-typing analysis.

### FTIR acquisition and analysis

The commercially available IR biotyper system (Bruker Daltonics GmbH, Bremen, Germany) is a FTIR spectroscopy-based technology which quantifies the absorption of infrared light from mainly (lipo)polysaccharides in a bacterial cell [15]. The molecular-specific vibrations are excited by absorption of light of a specific wavelength. Fast Fourier transformation of the detected signal, reduced in intensity due to absorption of the sample, leads to infrared spectra with characteristic absorption bands providing a fingerprint-like identification of the investigated sample mirroring the overall biochemical composition of cells [8–13].

According to the manufacturer’s instructions, two culture steps were performed to achieve stable growth and normal expression of the cell wall after storage at − 80 °C. All species were initially cultured on Colombia sheep blood agar (COS) plates, followed by subculturing on Trypticase soy agar (TSA) plates for *P. aeruginosa*, *A. baumannii/calcoaceticus complex* and *E. coli* and on COS for *S. aureus*, *E. faecium* and *E. faecalis*. The isolates were incubated for 24 (± 0.5) h at 37 °C in an normal air incubator. After the second culture step, bacterial suspensions were prepared in 1.5-ml vials containing 2 mm glass beads (VWR International B.V., Amsterdam, Netherlands) with a full 1 µl inoculation loop with bacteria in 50 µl of 70% ethanol. The bacterial suspension was thoroughly vortexed, after which 50 µl of sterile H_2_O was added. Then, 15 µl of the bacterial suspension was pipetted on a silicon plate in four technical replicates per isolate. Two Bruker Infrared Test Standard suspensions of 12 µl were added as controls. The plate was then dried at 37 °C in a normal air incubator for ± 20 min. All four technical replicates of isolates were analysed in each of three independent experiments on the IR biotyper system. Results were evaluated using Opus Software V7.5.18 and IR Biotyper Software V2.1.0.195 with the default settings (32 scans per technical replicate; spectral resolution, 6 cm^−1^; apodization function, Blackman-Harris 3-term; zero-filling factor 4). Following the manufacturer’s instructions, the pre-processing steps of the acquired data were as follows: the FTIR spectra measurements of individual technical replicates that did not meet the default quality criteria (0.4 < absorption < 2, signal-to-noise-ratio < 40, technical replicate of an isolate was not clustering with the other replicates of that isolate) were manually removed from further analysis to prevent inclusion of wrongful data. The FTIR spectra results of an entire isolate were removed when two technical replicates of this isolate did not meet the above criteria. The software based the infrared spectrum processing on the default setting of the manufacturer and analysed the wavenumber range of 800 to 1300 cm^−1^ of the spectra, measuring IR absorption of polysaccharides molecules in particular. The acquired FTIR spectra were analysed by hierarchical cluster analysis using Euclidean distance with average linkage criteria. The research analyst used these measures to manually select a cut-off for cluster identification of FTIR spectra. After removal of the aforementioned failed replicates, a cut-off was set based on the distance between the remaining replicates All these steps result in one FTIR run and were repeated three times to assess reproducibility. If reproducible, the overall hierarchical cluster analysis results of three FTIR repeated runs (each containing 4 technical replicates per isolate) were used for the comparison with the clustering results after whole genome sequencing (WGS) typing considered as the golden standard. If FTIR spectroscopy was not reproducible, FTIR results of the species were not considered for the comparison with WGS.

### Genome sequencing and assembly

QIAamp DNA mini kit (Cat. no. 51306) was used to extract genomic DNA following the manufacturer’s instructions. The DNA libraries were prepared with Illumina Nextera NA Flex Library Prep kit (Cat. No. 20018705) and Nextera DNA CD Indexes kit (Cat. No. 20018708) and sequenced with Illumina MiSeq Technology (Illumina, San Diego, CA, USA) using V2 chemistry and 150 bp paired-end settings. BioNumerics 8.0 (Applied Maths, Sint-Martens-Latem, Belgium) was used for WGS analysis including de novo genome assembly and whole genome MLST (wgMLST). Clustering was performed in BioNumerics using a minimum spanning tree algorithm based on wgMLST profiles. Clusters were defined as all profiles differ by less than 10 (for *E. faecium*, *E. faecalis* and *S. aureus*) or 20 (for *E. coli*, *P. aeruginosa* and *A. baumannii*) different alleles. Sequences were deposited in the European Nucleotide Archive (study accession number: PRJEB25629 and PRJEB51462).

### Congruence of cluster composition

The adjusted Rand index (ARI) was used to measure the reproducibility of FTIR typing. ARI indicates overall degree of equality of cluster composition between two FTIR spectroscopy repeated runs. An index of 1 indicates that the clusters of the first run show congruence of cluster composition with the clusters of second run. As we wanted to use FTIR spectroscopy as a rough first-line screening method, we argued the repeated runs should have at least an ARI of 0.7 for the inter-FTIR-assay results to be considered reproducible.

The adjusted Wallace index (AWI) was used to measure the congruence of cluster composition between FTIR and WGS typing. The AWI measures the directional probability that two isolates are clustered together by WGS and are also clustered together by FTIR spectroscopy. An index of 1 means all isolates clustered by WGS are also clustered by FTIR spectroscopy.

As the objective to assess FTIR spectroscopy as a real-time first-line screening for WGS, we argued that FTIR should at least cluster (almost) all isolates clustered by WGS in order not to miss any potential transmission (WGS → FTIR AWI of 0.95 or higher). Additionally, the AWI representing the directional probability of two isolates clustered together by FTIR spectroscopy was also clustered by WGS should in our opinion be 0.5 or higher (FTIR → WGS). This is to ensure an adequate discrimination of FTIR to filter for false-positive isolate clusters and not resulting in high costs due to unnecessary confirmatory WGS analysis. For the congruence of cluster composition analysis, we assumed that the current gold standard WGS is the most accurate test on cluster compositions. To calculate these two indices, we used the online tool http://www.comparingpartitions.info/ [16].

## Results

### Reproducibility of FTIR typing

The results of the FTIR repeated runs are summarized in Table 2 and the calculated ARIs in Table 3. The technical errors and removed measurements of the infrared absorption spectral measurements of *A. baumannii/calcoaceticus complex*, *E. coli* and *E. faecium* ranged from 0.5 to 15%, and more than 90% of the isolates grouped consistently in the same cluster for the repeated runs. Of the five tested species, the ARI for these three species had the highest congruence of cluster composition between the repeated runs, which ranged from 0.743 to 0.974. According to our chosen limit of 0.7, the ARIs indicated reproducibility. For *S. aureus*, the repeated runs had similar percentages of errors and removed measurements as the previously mentioned species, yet the isolates were not grouped as consistently. Less than 75% of the *S. aureus* isolates of run 2 or 3 were grouped in the same cluster as run 1. This was reflected in the ARI, which ranged from 0.446 to 0.615, and indicated the reproducibility of FTIR spectroscopy to be too low for S. aureus. FTIR results of the *P. aeruginosa* isolates showed the least favourable results; all three repeated runs had 25.8% or more technical errors, and at least 33.3% of the measurements were removed, resulting in few typeable isolates. Due to the lack of typeable isolates, no reliable ARI scores could be calculated for the FTIR spectroscopy repeated runs of P. aeruginosa.

**Table 2:** Amount of technical errors, removed measurements, typeability and isolates in same cluster in repeated runs of FTIRS-typing

Species	Number of measurements with technical error messages			Removed measurements from further analysis			Typeability of isolates			Typable isolate measurement in the same cluster compared to Run 1	
	Run 1	Run 2	Run 3	Run 1	Run 2	Run 3	Run 1	Run 2	Run 3	Run 2	Run 3
*A. baumannii*	3/100 (3.0%)	6/100 (6.0%)	2/100 (2.0%)	4/100 (4.0%)	11/100 (11.0%)	5/100 (5.0%)	24/25 (96.0%)	23/25 (92.0%)	24/25 (96.0%)	21/22 (95.5%)	21/23 (91.3%)
*E. coli*	3/124 (2.4%)	3/124 (2.4%)	1/124 (0.8%)	4/124 (3.2%)	3/124 (2.4%)	1/124 (0.8%)	30/31 (96.8%)	30/31 (96.8%)	31/31 (100%)	30/30 (100%)	28/30 (93.3%)
*E. faecium*	1/88 (1.1%)	3/88 (3.4%)	3/88 (3.4%)	0/88 (0.0%)	4/88 (4.5%)	5/88 (5.7%)	22/22 (100%)	21/22 (95.5%)	22/22 (100%)	20/21 (95.2%)	20/22 (90.9%)
*P. aeruginosa*	44/120 (36.7%)	31/120 (25.8%)	43/120 (35.8%)	51/120 (42.5%)	40/120 (33.3%)	45/120 (37.5%)	19/30 (63.3%)	21/30 (70.0%)	19/30 (63.3%)	16/16 (100%)	9/13 (69.2%)
*S. aureus*	7/148 (4.7%)	8/148 (5.4%)	13/148 (8.8%)	3/148 (2.0%)	8/148 (5.4%)	9/148 (6.1%)	37/37 (100%)	36/37 (97.3%)	37/37 (100%)	23/36 (63.9%)	27/37 (73.0%)

**Table 3:** Adjusted Rand index (ARI) comparing the FTIRS repeated runs

Species	Run 1 and 2	Run 1 and 3	Run 2 and 3
*A. baumannii*	0.854	0.864	0.743
*E. coli*	0.796	0.974	0.788
*E. faecium*	0.883	0.785	0.869
*S. aureus*	0.571	0.615	0.446

### Congruence of cluster composition of FTIR and WGS typing

As the FTIR spectroscopy analysis had no reproducibility for *S. aureus* and *P. aeruginosa*, the species were not included for the congruence of clustering between FTIR spectroscopy and WGS results.

The congruence of cluster composition analysis for both *A. baumannii* and *E. faecium* the WGS → FTIR AWI was higher than the argued limit of 0.95 (Table 4). This means that if two strains are in the same cluster by WGS, they have 95% chance or more of having the same FTIR cluster type. E. coli, however, did not meet the 0.95 threshold. The WGS → FTIR AWI for *E. coli* was 0.880. In the other direction of the AWI (FTIR → WGS), the results for all three species met the 0.5 threshold. This suggested FTIR spectroscopy had adequate discrimination to filter for false-positive isolate clusters and not lead to unnecessary confirmatory WGS analysis.

**Table 4:** Adjusted Wallace Index (AWI) of FTIRS- and WGS-typing^a^

Species	WGS → FTIRS	(95% CI)	FTIRS → WGS	(95% CI)
*A. baumannii*	1.000	(1.000–1.000)	0.986	(0.973–1.000)
*E. coli*	0.880	(0.811–0.949)	0.710	(0.436–0.985)
*E. faecium*	0.967	(0.934–1.000)	0.608	(0.292–0.924)

^a^The adjusted Wallace index is based on the raw data of 12 replicates per isolate

### Validation of FTIR typing as real-time screening method

The *E. faecalis* and *E. faecium* isolates were collected, because FTIR spectroscopy results of the *E. faecium* outbreak strains were reproducible and showed congruence with WGS. For 2 weeks, three isolates per ICU patient from routinely acquired rectal swab samples (for SDD surveillance) were collected, if growth of *E. faecium* and/or *E. faecalis* was detected. This resulted in 106 *E. faecalis* isolates of 31 patients and 104 *E. faecium* isolates of 29 patients. In Table 5, the technical errors and removed measurements according to the same default quality criteria as described earlier under “Methods”—FTIR spectroscopy acquisition and analysis—were summarized from the FTIR typing analysis on these *E. faecium* and *E. faecalis* collections. The FTIR typing results of the *E. faecalis* collection had more technical errors and measurements removed than *E. faecium*. Nonetheless, both the *E. faecalis* and *E. faecium* collections had an ARI above the chosen 0.7 reproducibility threshold (see Table 6).

**Table 5:** Total amount of technical errors and removed measurements of FTIRS typing of ICU isolates

Species	Number of measurements with technical error messages	Removed measurements from further analysis
*E. faecalis*	52/1272 (4.1%)	47/1272 (3.7%)
*E. faecium*	3/1248 (0,2%)	3/1248 (0,2%)

**Table 6:** Adjusted Rand index (ARI) comparing the FTIRS repeated runs of ICU isolates

Species	Run 1 and 2	Run 1 and 3	Run 2 and 3
*E. faecalis*	0.987	0.978	0.972
*E. faecium*	0.970	0.970	1.000

The FTIR spectroscopy analysis on the 106 *E. faecalis* isolates indicated 8 clusters. Isolates from four patients were clustered in two different FTIR spectroscopy clusters, and one patient had isolates in three different clusters. The largest cluster contained 14 different patients, followed by a cluster of 8 patients and a cluster of 7 different patients. Of the five remaining clusters, three contained two patients and two contained only one patient. The FTIR spectroscopy analysis on the 104 *E. faecium* isolates resulted in 12 different clusters. Isolates from 6 patients were clustered in two different FTIR clusters. The largest cluster contained 22 different patients, and of the remaining 6 clusters, only two clusters had 2 different patients, and the other 9 clusters contained just 1 patient. For both *E. faecalis* and *E. faecium* collections, the FTIR clusters showed no evident epidemiological link. Due to limited expenses, WGS was not applied to all isolates. Only one isolate per patient was randomly selected from *E. faecalis* FTIR cluster 1 of 7 patients and cluster 4 of 13 patients and *E. faecium* cluster 6 of 22 patients.

**Fig. 1:**
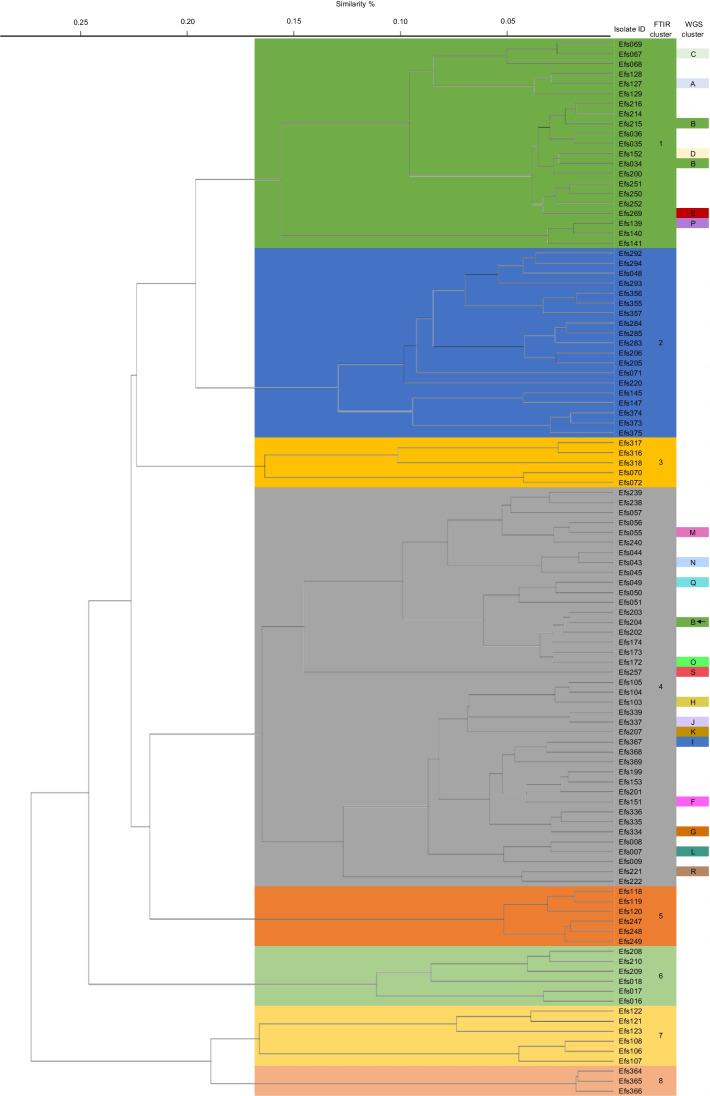
Clustering of FTIRS of 106 unrelated *E. faecalis*. The FTIRS clusters are displayed as shaded boxes. The corresponding WGS cluster of the isolate selected for analysis is displayed in the final column. Incongruently clustered isolates are marked with arrows

For *E. faecalis* 21 isolates from clusters 1 and 4 were selected for WGS typing. The WGS → FTIR AWI of 0.468 for *E. faecalis* suggests that the chance of two isolates clustered by WGS typing have less than 50% chance to cluster together by FTIR typing. The *E. faecalis* Isolate Efs 204 was placed in a different cluster by FTIR than WGS typing (see Fig. 1). Thus, suggesting screening with quick daily or weekly performed FTIR typing would have missed this isolate clustered by WGS. As expected, the more discriminatory method WGS typing subdivided the FTIR typing clusters of *E. faecalis* into smaller clusters. The FTIR cluster 1 of 7 patients resulted in 6 different WGS clusters and the FTIR cluster 4 of 14 patients resulted in 14 different WGS clusters. The FTIR → WGS AWI of 0.036 indicates that the chance of two isolates clustered by FTIR has a 3.6% chance to cluster together by WGS typing. This implies that FTIR typing has a too low discriminatory power to use as a screening method to detect potential outbreak clusters at patient wards and can result in unnecessary costly WGS confirmation.

**Fig. 2:**
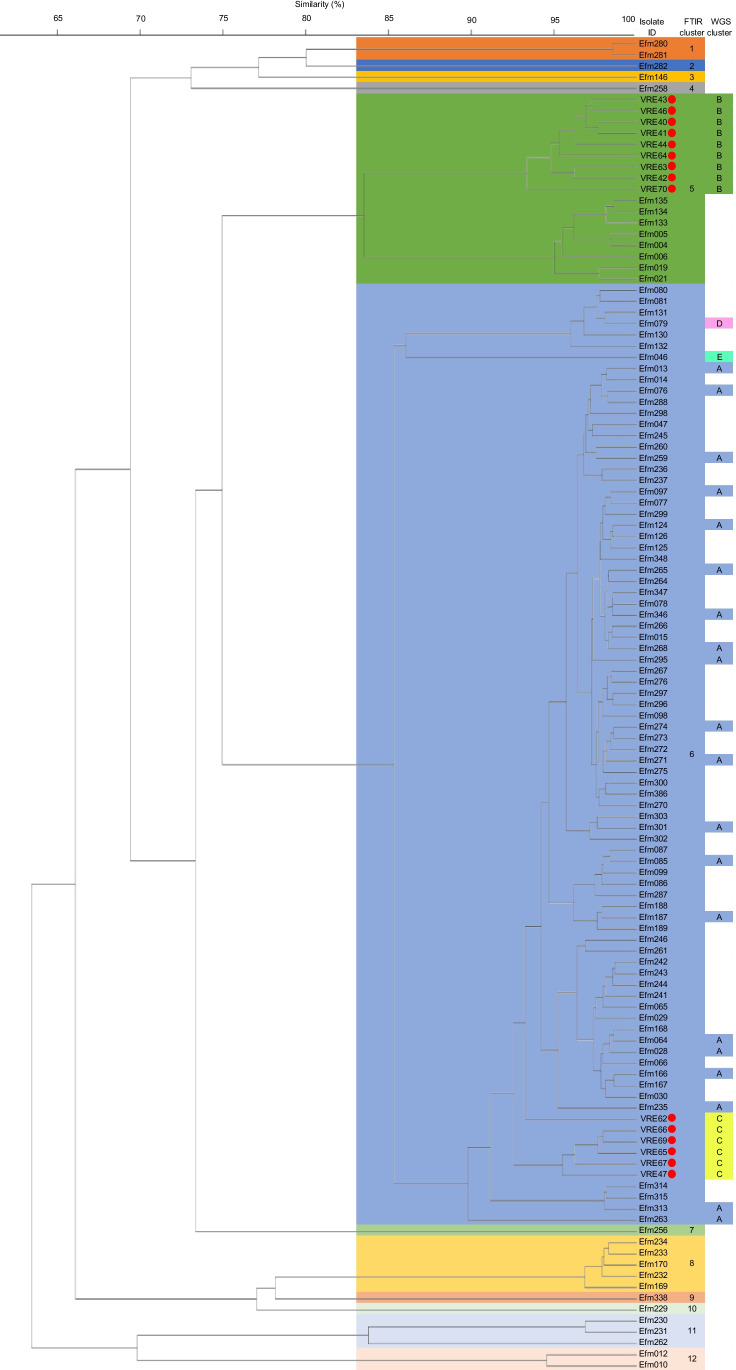
Clustering of FTIRS of 104 *E. faecium* collected without the presence of an evident outbreak and 15 vancomycin-resistant *E. faecium* (VRE) isolates of an outbreak. The VRE is indicated by a red circle. The FTIRS clusters are displayed as shaded boxes. The corresponding WGS cluster of the isolate selected for analysis is displayed in the final column

For *E. faecium*, 22 isolates from the largest cluster were selected for WGS typing. Because this was only one cluster, 15 outlier isolates from a recent VRE outbreak during first quarter of 2021 were added. The WGS → FTIR AWI of 1 for *E. faecium* indicates a 100% chance of all isolates clustered by WGS to be clustered together by FTIR spectroscopy. As with *E. faecalis*, the *E. faecium* FTIR → WGS AWI of 0.345 reflects that WGS typing is more discriminatory than FTIR typing. WGS separated the *E. faecium* isolates which were vancomycin resistant from the antibiotic-susceptible *E. faecium* isolates, whereas FTIR clustered them together (see Fig. 2). This FTIR → WGS AWI of 0.345 for a subset (*n* = 22) selected from a larger collection of *n* = 104 *E. faecium* routine sample isolates combined with a collection of *n* = 15 VRE outbreak isolates was substantially lower than the degree of congruence as measured by FTIR → WGS AWI for the well-characterized VRE outbreak collection of *n* = 22 isolates, as described in Table 4 (i.e., a FTIR → WGS AWI of 0.608) indicating the very limited discriminatory power of FTIR to use as a screening method to detect potential nosocomial transmission of *E. faecium* regardless of its antibiotic susceptibility profile at patient wards.

## Discussion

This study aims to assess if FTIR spectroscopy can function as an affordable real-time high-throughput screening method for hospital bacterial isolates, followed by WGS genotyping high-resolution confirmation, thus enabling rapid, reliable and cost-effective detection of the spread of bacteria.

This study considers FTIR spectroscopy suitable as real-time screening method if it meets the following three criteria. First, FTIR analysis should cluster at least most to all isolates clustered by WGS., we argued the congruence of cluster composition should have a WGS→FTIR AWI ≥ 0.95. Second, FTIR has to have adequate discrimination to filter for most false-positive isolate clusters to not result in unnecessary WGS analysis. We argued the congruence of cluster composition for FTIR→WGS AWI should be at least 0.5 or higher. And finally, FTIR spectroscopy results had to be reproducible. This was measured using the ARI, and we argued as an rough first-line method the ARI be 0.7 or higher. Unlike previous studies who used the ARI and AWI to compare methods [17–21], this study had set ARI and AWI limits for what was considered reproducible and congruence of cluster composition to reduce the subjectivity of the FTIR spectroscopy assessment per species. In order to minimize the influence of preferences of the researchers, these criteria were selected to ensure the objectivity of the research results on the assessment of FTIR per species. Hence, it is important to take into account that these limits were argued for the specific use of FTIR spectroscopy in this paper and are not universally used when using these two indices.

The results of the study are not altogether favourable. The congruence of cluster composition analysis is not conclusive whether FTIR spectroscopy clusters most to all isolates by WGS when used for routine samples. For the well-characterized outbreak isolate collections of the species *A. baumannii* and *E. faecium*, the WGS→FTIR AWI ≥ 0.95 criterion is met, as was the case for the subset of 22 of 104 *E. faecium* isolates plus 15 vancomycin resistant outbreak strains with a WGS→FTIR AWI of 1. However, when FTIR is applied to screen a large collection of isolates from routine samples, the cluster congruence may be too low. In the subset of 20 of the 106 *E. faecalis* isolates, FTIR did not detect all relevant clusters by WGS, and the WGS→FTIR AWI of 0.468 was too low according to the pre-set AWI limit. But given both AWI calculations were for a small subsection, it is unknown whether the AWI would be either higher or lower across the entire 106 or 104 isolates.

The second criterion is not met in this study: FTIR spectroscopy does not have adequate discrimination to filter for most false-positive isolate clusters when used for routine samples. For the well-characterized outbreak isolate collection of *A. baumannii*, *E. coli* and *E. faecium*, the FTIR→WGS AWI pre-set limit of 0.5 is met. However, this limit is not met when applied to a large collection of *E. faecalis* and *E. faecium* isolates from routine samples. FTIR spectroscopy clustered together large amounts of false-positive isolates for both species. In the *E. faecium* subset of 22 susceptible isolates and 15 vancomycin resistant isolates, FTIR spectroscopy was not able to discriminate between these isolates whereas WGS was. Additionally, the FTIR→WGS AWI decreased from 0.608 to 0.345 for the *E. faecium* collection of routine sample isolates. Two possible explanations for this difference in discriminatory power are the selection and number of isolates included in FTIR analysis. First, outbreak isolates are, unlike routine sample isolates, selected for genotyping analysis due to (multi-)resistance in antibiotic susceptibility reports. This selection already sieves through most of the isolates but misses potential clusters of antibiotic susceptible or wild-type isolates that are included when adding unbiased routine sample isolates. The sample selection influences the diversity in the population structure that is analysed and as a result influences the AWI. Typing methods compared using a species collection with high strain diversity in a large collection of isolates are less likely to be congruent than a species collection consisting of only two or three clonal complexes. Hence, the results of our sample collections need to be interpreted with care for another hospital which has a different isolate collection method. Second, the inclusion of susceptible isolates increases the sample size for FTIR analysis and could change the subjective interpretation of which isolates are considered similar or different according to the laboratory analyst, as there is no pre-set similarity cut-off established yet for FTIR spectroscopy.

Although FTIR spectroscopy is reproducible and thus the third criterion is met, the laboratory process and analysis is difficult to standardize. For both the well-characterized outbreak isolates and the isolates from routine samples, the repeated runs of FTIR had ARIs higher than 0.7. This indicates FTIR spectroscopy repeated runs were reproducible, which is in line with previous studies [13, 22]. However, despite the rigorous standard operating procedure, during the acquisition of the FTIR data, it was observed that the quality of the infrared absorption are strongly influenced by small difference in culture media, incubation time and processing of aqueous samples, as mentioned in previous studies [2, 9]. This influence is reflected in the lack of reproducibility of *S. aureus* on a solid medium (i.e., COS, blood agar), but two previous studies concluded FTIR spectroscopy is reliable for S. aureus in liquid media [12, 23]. Another study by Martak et al. [19] concluded FTIR spectroscopy to be successful for *P. aeruginosa*, but not for *A. baumannii*. Unlike our study, they only applied one incubation time of 24 h before creating the bacterial suspension. A study by Hu et al. [24] noticed, like this study, that the official method for bacterial suspension, even after extended vortexing, a stable and homogenous suspension could not be formed for all samples. The suspension quality influenced the successful acquisition rates, and Hu et al. had to modify their sample preparation method to gain better results. This further reflects FTIR spectroscopy sensitivity to variation of sample preparation and how this can influence the quality of infrared absorption. Furthermore, as mentioned before, the analysis is subjective to the interpretation of the laboratory analyst due to the lack of a pre-set similarity cut-off for isolate clustering. Hence, the cluster analysis could differ per analyst, and thus the FTIR spectroscopy cluster results. A solution to reduce subjectivity in the FTIR analysis could be setting a standard similarity cut-off per species.

Rapid, reliable and inexpensive bacterial typing methods can have a great impact on the quick detainment of outbreaks, and therefore, they can be useful for reducing a considerable health and financial burden. For well-characterized and selected on the basis of multi-resistance outbreak isolates of *A. baumannii/calcoaceticus complex* and *E. faecium*, FTIR spectroscopy appears to be a powerful discriminatory typing tool as was also shown for *Klebsiella pneumoniae* by Rakovitsky et al. [25]. However, our study shows that the discriminatory power of FTIR spectroscopy is too low to screen real-time and unbiased for potential transmission of *E. faecium* and *E. faecalis* at patient wards based on isolates acquired in routine surveillance cultures when there is no clear suspicion of an ongoing outbreak with (multi-)resistant bacteria. Nevertheless, this is, to our knowledge, the first study to assess the use of FTIR spectroscopy as a rapid screening tool to detect potential clonal clusters in routine sample isolates. Therefore, more studies are needed to assess FTIR spectroscopy as a real-time screening method for potential clonal clusters in routine sample isolates of also other bacterial species than *E. faecalis* and *E. faecium*. If FTIR spectroscopy can be standardized on both the wetlab process and the cluster analysis, it might become an attractive typing tool.

## Supplementary Information

Below is the link to the electronic supplementary material.

Supplementary file1 (PDF 288 KB)

## Abbreviations

AFLP: Amplified fragment length polymorphism
WGS: Whole genome sequencing
FTIR: Fourier transform infrared spectroscopy
ICU: Intensive care units
SDD: Selective decontamination of the digestive tract
COS: Colombia sheep blood agar
TSA: Trypticase soy agar
ARI: Adjusted rand index
AWI: Adjusted Wallace index
