# Supplementary material for: Comparison of fast Fourier transform infrared spectroscopy biotyping with whole genome sequencing-based genotyping in common nosocomial pathogens
Source: Anal Bioanal Chem. 2022 Aug 13;414(24):7179–89. doi: 10.1007/s00216-022-04270-6 (PMC9482911; doi:10.1007/s00216-022-04270-6)
Supplement: Supplementary file 1 — Supplementary file1 (PDF 288 KB) [file 216_2022_4270_MOESM1_ESM.pdf]

Supplement A.1. All available characteristics of the 25 Acinetobacter Baumannii isolates included in either FTIRS, WGS or both analysis methods.

Study number	Year of isolation	Material of isolation	Antibiogram				
			Ciprofloxacin	Gentamicine	Imipenem	Meropenem	Tobramycine
ACE 01	2015	NA	NA	NA	NA	NA	NA
ACE 02	2015	NA	NA	NA	NA	NA	NA
ACE 03	2015	NA	NA	NA	NA	NA	NA
ACE 04	2015	NA	NA	NA	NA	NA	NA
ACE 05	2018	Clinical	R	R	R	R	R
ACE 06	2018	Clinical	R	R	R	R	R
ACE 07	2018	Clinical	R	R	R	R	R
ACE 08	2018	Clinical	R	R	R	R	R
ACE 09	2018	Clinical	R	R	R	R	R
ACE 10	2018	Environment	S	S	S	S	S
ACE 11	2018	Environment	S	S	S	S	S
ACE 12	2018	Environment	S	S	S	S	S
ACE 13	2018	Environment	S	S	S	S	S
ACE 14	2018	Clinical	R	R	R	R	R
ACE 15	2018	Clinical	R	R	R	R	R
ACE 16	2018	Clinical	R	R	R	R	R
ACE 17	2018	Clinical	R	R	R	R	R
ACE 18	2018	Clinical	R	R	R	R	R
ACE 19	2018	Environment	R	R	R	R	R
ACE 20	2018	Environment	R	R	R	R	R
ACE 21	2018	Environment	R	R	R	R	R
ACE 22	2018	Environment	R	R	R	R	R
ACE 23	2018	Environment	NA	NA	NA	NA	NA
ACE 24	2018	Environment	NA	NA	NA	NA	NA
ACE 27	2018	Clinical	R	S	R	R	S

R = Resistant S = Susceptible I = Susceptible at increased exposure NA = Not Available

Supplement A.2. All available characteristics of the 31 Escherichia coli isolates included in either FTIRS, WGS or both analysis methods.

Study number	Year of isolation	Material of isolation	Antibiogram								
			ESBL	Ceftriaxone	Ceftazidim	Cefotaxim	Ciprofloxacin	Gentamicine	Imipenem	Meropenem	Tobramycine
ECO 01	2014	Clinical	-	S	S	S	S	S	S	S	S
ECO 02	2014	Clinical	-	S	S	S	S	S	S	S	S
ECO 03	2014	Clinical	-	S	S	S	S	S	S	S	S
ECO 04	2015	Clinical	+	R	R	R	S	S	S	S	S
ECO 05	2015	Clinical	+	R	R	R	S	S	NA	S	S
ECO 06	2015	Clinical	+	R	NA	R	R	S	NA	S	NA
ECO 07	2015	Clinical	+	R	NA	R	R	S	NA	S	NA
ECO 08	2015	Clinical	+	R	NA	R	R	S	NA	S	NA
ECO 09	2015	Clinical	+	R	NA	R	R	S	NA	S	NA
ECO 10	2015	Clinical	+	NA	NA	NA	NA	NA	NA	NA	NA
ECO 11	2015	NA	+	NA	NA	NA	NA	NA	NA	NA	NA
ECO 12	2015	Clinical	+	R	NA	R	R	S	NA	S	NA
ECO 13	2016	Clinical	-	S	NA	S	R	R	NA	NA	R
ECO 14	2016	Clinical	-	S	NA	S	R	R	NA	NA	R
ECO 15	2016	Clinical	-	S	NA	S	S	S	NA	NA	NA
ECO 16	2016	Clinical	-	S	NA	S	S	S	NA	NA	NA
ECO 17	2017	Clinical	-	S	S	S	R	R	S	S	R
ECO 18	2017	Clinical	-	R	R	R	R	R	S	S	R
ECO 19	2017	Clinical		R	R	R	R	S	S	S	S
ECO 20	2017	Clinical	+	R	R	R	R	R	S	S	R
ECO 21	2017	Clinical	+	R	R	R	R	R	S	S	R
ECO 22	2018	Clinical	+	R	R	R	R	R	S	S	R
ECO 23	2019	Clinical	-	R	R	R	R	R	R	I	R
ECO 24	2019	Clinical	-	R	R	R	S	S	R	R	S
ECO 25	2019	Clinical	-	R	R	R	R	S	R	R	S

ECO 26	2019	Clinical	-	R	R	R	S	S	I	I	S
ECO 27	2019	Clinical	-	R	R	R	S	S	I	I	S
ECO 28	2019	Clinical	-	R	R	R	S	S	R	I	S
ECO 29	2018	Clinical	-	R	R	R	R	S	R	R	S
ECO 30	2018	Clinical	-	R	R	R	R	R	R	I	R
ECO 31	2018	Clinical	-	R	R	R	R	R	R	R	R

R = Resistant S = Susceptible I = Susceptible at increased exposure NA = Not Available + = Extended-Spectrum Beta-Lactamases (ESBL) positive - = ESBL negative

Supplement A.3. All available characteristics of the 22 Enterococcus faecium isolates included in either FTIRS, WGS or both analysis methods.

Study number	Year of isolation	Material of isolation	Antibiogram
VRE 02	2017	Clinical	VRE
VRE 05	2017	Clinical	VRE
VRE 13	2017	Clinical	VRE
VRE 14	2017	Clinical	VRE
VRE 15	2017	Clinical	VRE
VRE 16	2017	Clinical	VRE
VRE 17	2017	Clinical	VRE
VRE 18	2017	Clinical	VRE
VRE 19	2017	Clinical	VRE
VRE 20	2017	Clinical	VRE
VRE 21	2017	Clinical	VRE
VRE 28	2017	Clinical	VRE
VRE 30	2019	NA	VRE
VRE 31	2020	NA	VRE
VRE 32	2019	NA	VRE
VRE 33	2019	NA	VRE
VRE 34	2019	NA	VRE
VRE 35	2019	NA	VRE

VRE 36	2019	NA	VRE
VRE 37	2019	NA	VRE
VRE 38	2019	NA	VRE
VRE 39	2019	NA	VRE

VRE = Vancomycin resistant *Enterococcus faecium*

Supplement A.4. All available characteristics of the 37 Staphylococcus aureus isolates included in either FTIRS, WGS or both analysis methods.

Study number	Year of isolation	Material of isolation	Antibiogram
SAU 01	2015	Clinical	MRSA
SAU 02	2015	Clinical	MSSA
SAU 03	2015	Clinical	MRSA
SAU 04	2015	Clinical	MSSA
SAU 05	2015	Clinical	MRSA
SAU 06	2015	Clinical	MRSA
SAU 07	2016	Clinical	MRSA
SAU 08	2016	Clinical	MRSA
SAU 10	2017	Clinical	MRSA
SAU 11	2017	Clinical	MRSA
SAU 12	2017	Clinical	MRSA
SAU 13	2018	Clinical	MSSA
SAU 14	2018	Clinical	MSSA
SAU 15	2018	Clinical	MSSA
SAU 16	2018	Clinical	MRSA
SAU 17	2018	Clinical	MRSA
SAU 18	2018	Clinical	MRSA
SAU 19	2018	Clinical	MRSA
SAU 20	2018	Clinical	MRSA
SAU 21	2018	Clinical	MSSA
SAU 22	2018	Clinical	MRSA

SAU 23	2018	Clinical	MRSA
SAU 24	2018	Clinical	MRSA
SAU 25	2018	Clinical	MRSA
SAU 26	2019	Clinical	MRSA
SAU 27	2019	Clinical	MSSA
SAU 28	2019	Clinical	MSSA
SAU 29	2019	Clinical	MSSA
SAU 30	2019	Clinical	MRSA
SAU 31	2019	Clinical	MRSA
SAU 32	2019	Clinical	MRSA
SAU 33	2019	Clinical	MSSA
SAU 34	2019	Clinical	MSSA
SAU 35	2019	NA	MSSA
SAU 36	2019	NA	MSSA
SAU 37	2019	Clinical	MRSA
SAU 38	2019	Clinical	MRSA

MSSA = Methicillin-susceptible *Staphylococcus aureus*

MRSA = Methicillin-resistant *Staphylococcus aureus*

Supplement A.5. All available characteristics of the 30 Pseudomonas aeruginosa isolates included in either FTIRS, WGS or both analysis methods.

Study number	Year of isolation	Material of isolation	Antibiogram					
			Ceftazidim	Ciprofloxacin	Imipenem	Meropenem	Tobramycine	Piperacilline
PSA 01	NA	NA	NA	NA	NA	NA	NA	NA
PSA 02	2011	NA	NA	NA	NA	NA	NA	NA
PSA 03	2011	NA	NA	NA	NA	NA	NA	NA
PSA 04	2011	NA	NA	NA	NA	NA	NA	NA
PSA 05	2011	NA	NA	NA	NA	NA	NA	NA
PSA 06	2011	NA	NA	NA	NA	NA	NA	NA
PSA 07	2011	NA	NA	NA	NA	NA	NA	NA

PSA 08	2011	NA	NA	NA	NA	NA	NA	NA
PSA 09	2014	NA	NA	NA	NA	NA	NA	NA
PSA 10	2014	NA	NA	NA	NA	NA	NA	NA
PSA 11	2014	NA	NA	NA	NA	NA	NA	NA
PSA 12	2014	NA	NA	NA	NA	NA	NA	NA
PSA 13	2014	Environment	R	R	R	R	R	NA
PSA 14	2014	Clinical	R	R	R	R	R	R
PSA 15	2015	Environment	NA	NA	NA	NA	NA	NA
PSA 16	2016	Clinical	R	R	R	R	R	NA
PSA 17	2016	Clinical	I	R	R	R	R	NA
PSA 18	2016	Clinical	R	R	R	R	R	R
PSA 19	2016	Environment	NA	NA	NA	NA	NA	NA
PSA 20	2016	Environment	NA	NA	NA	NA	NA	NA
PSA 21	2017	Clinical	R	R	R	R	R	NA
PSA 22	2017	Clinical	R	R	R	R	R	NA
PSA 23	2018	Environment	NA	NA	NA	NA	NA	NA
PSA 24	2018	Environment	NA	NA	NA	NA	NA	NA
PSA 25	2018	Environment	NA	NA	NA	NA	NA	NA
PSA 26	2018	Clinical	R	R	I	R	S	NA
PSA 27	2019	Clinical	S	S	S	S	S	NA
PSA 28	2019	Clinical	S	S	S	S	S	NA
PSA 29	2019	Clinical	S	S	S	S	S	NA
PSA 30	2019	Clinical	R	R	I	I	S	NA

R = Resistant S = Susceptible I = Susceptible at increased exposure NA = Not Available

FTIRS cluster of 22 *E. faecium* isolates

Isolate ID	Reprun 1	Reprun 2	Reprun 3
VRE 02	Cluster 01	Cluster 01	Removed
VRE 05	Cluster 01	Cluster 01	Cluster 01
VRE 13	Cluster 01	Cluster 01	Cluster 01
VRE 14	Cluster 01	Cluster 01	Cluster 01
VRE 15	Cluster 01	Cluster 07 ←	Cluster 03 ←
VRE 16	Cluster 01	Cluster 01	Cluster 01
VRE 17	Cluster 01	Cluster 01	Cluster 01
VRE 18	Cluster 01	Cluster 01	Cluster 01
VRE 19	Cluster 01	Removed	Cluster 01
VRE 20	Cluster 01	Cluster 01	Cluster 01
VRE 21	Cluster 01	Cluster 01	Cluster 03 ←
VRE 28	Cluster 01	Cluster 01	Cluster 01
VRE 38	Cluster 01	Cluster 01	Cluster 01
VRE 30	Cluster 02	Cluster 02	Cluster 02
VRE 31	Cluster 02	Cluster 02	Cluster 02
VRE 32	Cluster 02	Cluster 02	Cluster 02
VRE 33	Cluster 02	Cluster 02	Cluster 02
VRE 34	Cluster 02	Cluster 02	Cluster 02
VRE 35	Cluster 02	Cluster 02	Cluster 02
VRE 36	Cluster 04	Cluster 04	Cluster 04
VRE 37	Cluster 05	Cluster 05	Cluster 05
VRE 39	Cluster 06	Cluster 06	Cluster 06

FTIRS cluster of 25 *A. baumannii* isolates

Isolate ID	Reprun 1	Reprun 2	Reprun 3
ACE 6	Cluster 01	Cluster 01	Cluster 01
ACE 14	Cluster 01	Cluster 01	Cluster 01
ACE 15	Cluster 01	Cluster 01	Cluster 01
ACE 16	Cluster 01	Cluster 02 ←	Cluster 01
ACE 17	Cluster 01	Cluster 01	Cluster 01
ACE 18	Cluster 01	Cluster 01	Cluster 01
ACE 19	Cluster 01	Cluster 01	Cluster 01
ACE 20	Cluster 01	Cluster 01	Cluster 01
ACE 21	Cluster 01	Cluster 01	Cluster 01
ACE 22	Cluster 01	Cluster 01	Cluster 01
ACE 23	Cluster 01	Cluster 01	Cluster 01
ACE 24	Cluster 01	Cluster 01	Cluster 01
ACE 5	Cluster 02	Cluster 02	Cluster 02
ACE 7	Cluster 02	Cluster 02	Cluster 02
ACE 8	Cluster 02	Cluster 02	Cluster 01 ←
ACE 9	Cluster 02	Removed	Cluster 02
ACE 10	Cluster 03	Cluster 03	Cluster 03
ACE 12	Cluster 03	Cluster 03	Cluster 03
ACE 13	Cluster 03	Removed	Cluster 03
ACE 11	Removed	Cluster 03	Cluster 03
ACE 1	Cluster 04	Cluster 04	Cluster 04
ACE 4	Cluster 04	Cluster 04	Cluster 04
ACE 2	Cluster 05	Cluster 05	Cluster 07 ←
ACE 3	Cluster 05	Cluster 05	Removed
ACE 27	Cluster 06	Cluster 06	Cluster 06

FTIRS cluster of 31 *E. coli* isolates

Isolate ID	Reprun 1	Reprun 2	Reprun 3
ECO1	Cluster 01	Cluster 01	Cluster 01
ECO2	Cluster 01	Cluster 01	Cluster 01
ECO3	Cluster 01	Cluster 01	Cluster 01
ECO4	Cluster 02	Cluster 02	Cluster 02
ECO5	Cluster 02	Cluster 02	Cluster 02
ECO6	Cluster 03	Cluster 03	Cluster 03
ECO8	Cluster 03	Cluster 03	Cluster 03
ECO10	Cluster 03	Cluster 03	Cluster 03
ECO12	Cluster 03	Cluster 03	Cluster 03
ECO20	Cluster 03	Cluster 03	Cluster 03
ECO11	Cluster 04	Cluster 04	Cluster 04
ECO16	Cluster 04	Cluster 04	Cluster 04
ECO26	Cluster 05	Cluster 05	Cluster 05
ECO27	Cluster 05	Cluster 05	Cluster 05
ECO28	Cluster 05	Cluster 05	Cluster 05
ECO17	Cluster 06	Cluster 06	Cluster 06
ECO24	Cluster 06	Cluster 06	Cluster 06
ECO18	Removed	Removed	Cluster 06
ECO7	Cluster 07	Cluster 07	Cluster 03 ←
ECO9	Cluster 07	Cluster 07	Cluster 03 ←
ECO25	Cluster 08	Cluster 08	Cluster 08
ECO30	Cluster 08	Cluster 08	Cluster 08
ECO13	Cluster 09	Cluster 09	Cluster 09
ECO14	Cluster 10	Cluster 10	Cluster 10
ECO15	Cluster 11	Cluster 11	Cluster 11
ECO19	Cluster 12	Cluster 12	Cluster 12
ECO21	Cluster 13	Cluster 13	Cluster 13
ECO22	Cluster 14	Cluster 14	Cluster 14
ECO23	Cluster 15	Cluster 15	Cluster 15
ECO29	Cluster 16	Cluster 16	Cluster 16
ECO31	Cluster 17	Cluster 17	Cluster 17

FTIRS cluster of 37 *S. aureus* isolates

Isolate ID	Reprun 1	Reprun 2	Reprun 3
SAU 02	Cluster 01	Cluster 01	Cluster 01
SAU 03	Cluster 01	Cluster 01	Cluster 19 ←
SAU 04	Cluster 01	Cluster 01	Cluster 01
SAU 14	Cluster 01	Cluster 01	Cluster 01
SAU 15	Cluster 01	Cluster 01	Cluster 01
SAU 28	Cluster 01	Cluster 01	Cluster 03 ←
SAU 29	Cluster 01	Cluster 01	Cluster 01
SAU 30	Cluster 01	Cluster 01	Cluster 01
SAU 34	Cluster 01	Cluster 01	Cluster 01
SAU 35	Cluster 01	Cluster 01	Cluster 01
SAU 36	Cluster 01	Cluster 01	Cluster 01
SAU 06	Cluster 02	Cluster 02	Cluster 02
SAU 07	Cluster 02	Cluster 16 ←	Cluster 02
SAU 08	Cluster 02	Cluster 06 ←	Cluster 02
SAU 09	Cluster 02	Cluster 06 ←	Cluster 02
SAU 10	Cluster 02	Cluster 02	Cluster 02
SAU 11	Cluster 02	Cluster 02	Cluster 02
SAU 12	Cluster 02	Cluster 02	Cluster 02
SAU 16	Cluster 02	Cluster 02	Cluster 02
SAU 17	Cluster 02	Cluster 02	Cluster 02
SAU 26	Cluster 02	Cluster 02	Cluster 02
SAU 27	Cluster 02	Cluster 02	Cluster 20 ←
SAU 38	Cluster 02	Cluster 17 ←	Cluster 02
SAU 23	Cluster 03	Cluster 18 ←	Cluster 03
SAU 24	Cluster 03	Removed	Cluster 03
SAU 18	Cluster 05	Cluster 01 ←	Cluster 21 ←
SAU 19	Cluster 05	Cluster 01 ←	Cluster 01 ←
SAU 01	Cluster 06	Cluster 06	Cluster 06
SAU 05	Cluster 07	Cluster 03 ←	Cluster 03 ←
SAU 13	Cluster 08	Cluster 08	Cluster 08
SAU 20	Cluster 09	Cluster 09	Cluster 01 ←
SAU 21	Cluster 10	Cluster 10	Cluster 10
SAU 22	Cluster 11	Cluster 04 ←	Cluster 11
SAU 25	Cluster 12	Cluster 01 ←	Cluster 12
SAU 31	Cluster 13	Cluster 04 ←	Cluster 01 ←
SAU 32	Cluster 14	Cluster 03 ←	Cluster 03 ←
SAU 33	Cluster 15	Cluster 01 ←	Cluster 01 ←

FTIRS cluster of 30 *P. aeruginosa* isolates

Isolate ID	Reprun 1	Reprun 2	Reprun 3
PSA 01	Cluster 03	Cluster 03	Cluster 02 ←
PSA 02	Cluster 04	Cluster 02 ←	Cluster 01 ←
PSA 03	Cluster 05	Cluster 05	Cluster 05
PSA 04	Cluster 06	Removed	Cluster 06
PSA 05	Cluster 07	Cluster 07	Cluster 07
PSA 06	Cluster 08	Cluster 08	Cluster 08
PSA 07	Cluster 09	Cluster 09	Cluster 02 ←
PSA 08	Cluster 10	Removed	Cluster 02 ←
PSA 09	Cluster 11	Cluster 11	Cluster 11
PSA 10	Removed	Cluster 12	Cluster 12
PSA 11	Cluster 13	Cluster 13	Removed
PSA 12	Cluster 14	Cluster 14	Cluster 14
PSA 13	Removed	Removed	Removed
PSA 14	Removed	Cluster 01	Removed
PSA 15	Removed	Cluster 01	Cluster 01
PSA 16	Removed	Removed	Cluster 01
PSA 17	Removed	Removed	Removed
PSA 18	Removed	Removed	Cluster 01
PSA 19	Cluster 15	Removed	Removed
PSA 20	Cluster 16	Cluster 16	Removed
PSA 21	Removed	Removed	Removed
PSA 22	Removed	Removed	Removed
PSA 23	Cluster 17	Cluster 17	Removed
PSA 24	Cluster 18	Cluster 18	Cluster 18
PSA 25	Cluster 19	Cluster 19	Cluster 19
PSA 26	Cluster 20	Cluster 20	Cluster 20
PSA 27	Removed	Cluster 23	Removed
PSA 28	Cluster 21	Cluster 21	Cluster 21
PSA 29	Removed	Cluster 24	Cluster 24
PSA 30	Cluster 22	Cluster 22	Removed

Supplement B. FTIRS cluster analysis of three replicate runs of A. baumannii, E. coli, E. faecium, S. aureus and P. aeruginosa isolates. The FTIRS clusters of reprun 1 are displayed as shaded boxes. The arrows indicate incongruently clustered isolates between rep run 1 compared to rep run 2 or 3.

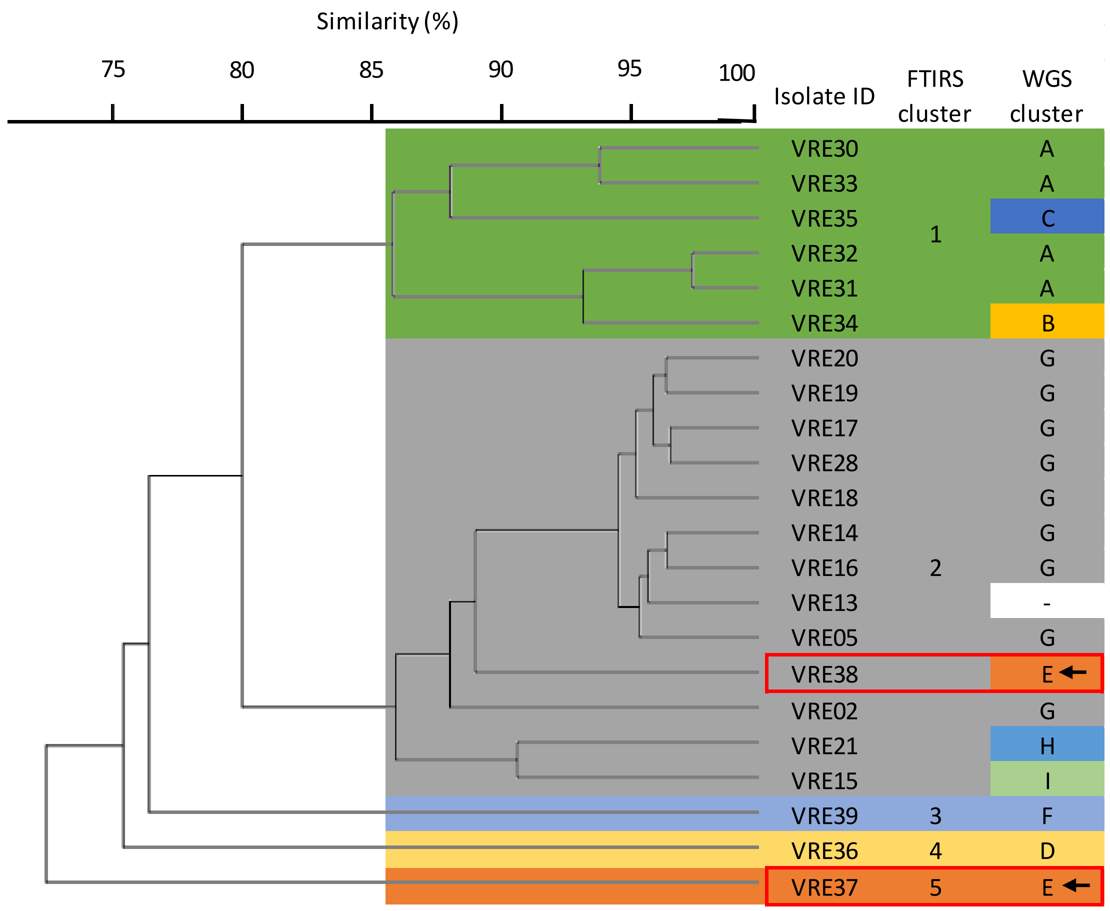

Supplement C.1. Clustering of FTIRS of 22 E. faecium isolates and the corresponding WGS and AFLP cluster of the isolate[a]. FTIRS clusters are displayed as shaded boxes. Incongruently clustered isolates are marked with arrows.

[a]Due to the size of the figure, the mean of the 12 replicates per isolate was used to display the tree instead of the raw data per isolate.

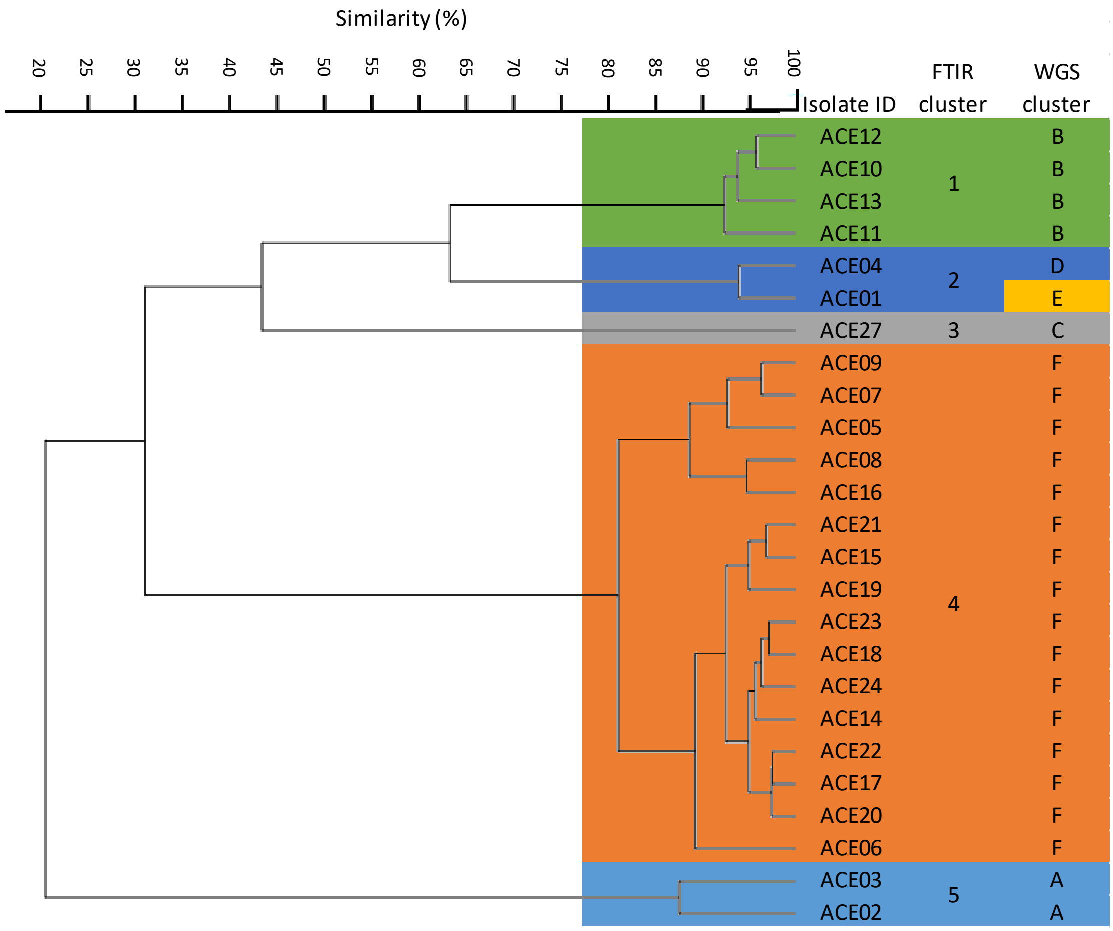

Supplement C.2. Clustering of FTIRS of 25 A. baumannii isolates and the corresponding WGS and AFLP cluster of the isolate[a]. FTIRS clusters are displayed as shaded boxes.

[a]Due to the size of the figure, the mean of the 12 replicates per isolate was used to display the tree instead of the raw data per isolate.

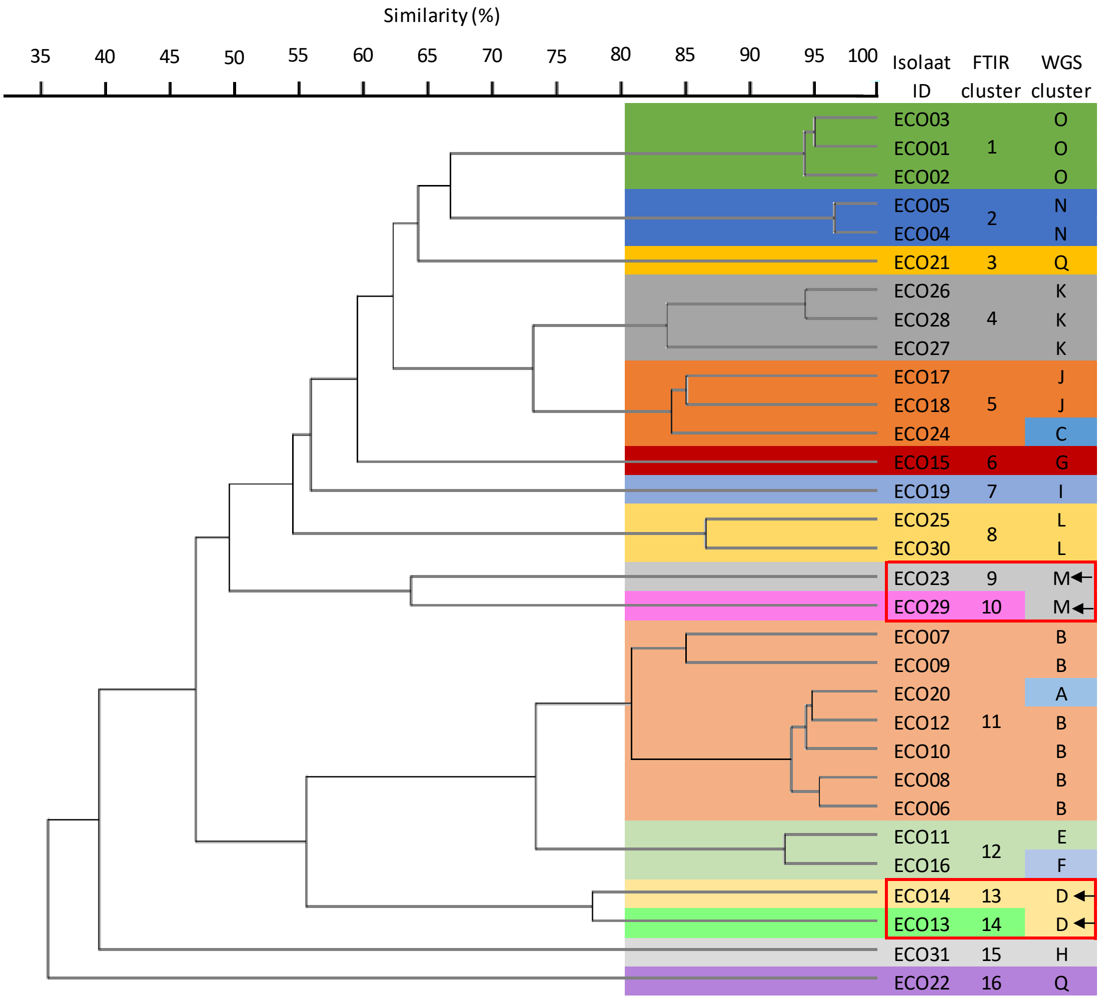

Supplement C.3. Clustering of FTIRS of 31 E. coli isolates and the corresponding WGS and AFLP cluster of the isolate[a]. FTIRS clusters are displayed as shaded boxes. Incongruently clustered isolates are marked with arrows.

[a]Due to the size of the figure, the mean of the 12 replicates per isolate was used to display the tree instead of the raw data per isolate.
